# Automated sorting of neuronal trees in fluorescent images of neuronal networks using NeuroTreeTracer

**DOI:** 10.1038/s41598-018-24753-w

**Published:** 2018-04-24

**Authors:** Cihan Kayasandik, Pooran Negi, Fernanda Laezza, Manos Papadakis, Demetrio Labate

**Affiliations:** 1 University of Houston, Department of Mathematics, Houston, Texas United States of America; 2 University of Texas Medical Branch, Department of Pharmacology and Toxicology, Galveston, Texas United States of America

## Abstract

Fluorescence confocal microscopy has become increasingly more important in neuroscience due to its applications in image-based screening and profiling of neurons. Multispectral confocal imaging is useful to simultaneously probe for distribution of multiple analytes over networks of neurons. However, current automated image analysis algorithms are not designed to extract single-neuron arbors in images where neurons are not separated, hampering the ability map fluorescence signals at the single cell level. To overcome this limitation, we introduce NeuroTreeTracer – a novel image processing framework aimed at automatically extracting and sorting single-neuron traces in fluorescent images of multicellular neuronal networks. This method applies directional multiscale filters for automated segmentation of neurons and soma detection, and includes a novel tracing routine that sorts neuronal trees in the image by resolving network connectivity even when neurites appear to intersect. By extracting each neuronal tree, NeuroTreetracer enables to automatically quantify the spatial distribution of analytes of interest in the subcellular compartments of individual neurons. This software is released open-source and freely available with the goal to facilitate applications in neuron screening and profiling.

## Introduction

Neuronal reconstruction is critical in a variety of neurobiological studies. During the last two decades, a large number of algorithms and software toolkits were developed aiming at providing digital reconstruction of neurons from images acquired using bright field or fluorescent microscopy^1^. Without attempting to provide a complete list of relevant publications, we recall that existing automated or semi-automated methods include several academic routines^2–7^ and other freeware (e.g., Neuromantic^8^, Neuronstudio^9,10^) or commercial software packages (e.g., Imaris^11^, Neurolucida^12^, PerkinElmer Cellular Imaging^13^) offering multiple capabilities. Many current methods still require significant manual intervention^1,14^ and their performance is typically very sensitive on the types of data. Yet a huge effort is under way in the scientific community to create fully automated algorithms for problems of neuronal reconstruction. The impact of these methods in neuroscience is expected to be very significant. Digital reconstruction algorithms have the potential to extract multiple morphometric parameters, facilitating the statistical analysis and formulation of computational models designed to predict structural changes induced by genetic or chemical perturbations.

Tracing is an especially critical task in neuronal reconstruction as it provides the backbone for building a geometrical representation of neurons. A major effort in improving neuronal tracing and reconstruction emerged during the last decade in response to the DIADEM Challenge^15,16^ and, more recently, as part of the ambitious BigNeuron project^17^. As a result of these efforts, several powerful algorithms were proposed delivering robust and accurate neuronal tracing^2–7^. Nevertheless, these methods are either aimed at processing single-neuron images or designed to trace any structure in an image *without sorting neurites into arbors corresponding to individual neurons and without resolving neuronal connectivity*. Available high-content screening software such as the HCA-Vision^18^, for instance, can efficiently trace an image of a multicellular network and extract several morphometric characteristics. However, this method is not designed to correctly identify single-neuron traces unless the neurons are well separated, nor to identify the path connecting a soma to a specific neurite location. The inability to automatically sort traces into individual neuronal arbors is a significant limitation in image-based applications of neuron screening and profiling where it is required to measure local expression levels of molecular constituents of axons and dendrites relative to their respective cells.

Extracting individual neuronal traces from an image of a multicellular network is a challenging task, in general, even when the entire image has been traced, due to the need to separate vessels that appear to cross or run very close to each other, and the need to resolve the connectivity of a networks that can be topologically complex (what path leads from a neurite location to its soma?). To address this limitation and facilitate the automated collection of local fluorescent expression measures from individual neurons in a network, in this paper we introduce a novel neurite tracing and sorting algorithm, called NeuroTreeTracer, designed to identify and trace individual neuronal trees in 2-dimensional fluorescent images of networks containing multiple (non-separated) neurons. Our method is tailored to the needs of confocal images of neuronal cultures – a technique that provides a well controlled setting to study critical properties of neurons in near physiological conditions and that is commonly used in applications of neuron screening and profiling.

Automatically resolving the topological structure in a confocal image of a neuronal culture can be particularly challenging. Such data typically consist of stacks containing 10–20 images so that only 10–20 pixels are available along the *z*-direction as compared with the *x* and *y* directions where length can be 512 pixels or more. As a consequence, the ‘data cube’ is very thin along one of its axes. Furthermore, due to the acquisition process, the image contrast degrades rapidly as optical slices are further away from the light source. As a result, neurites belonging to different neurons may appear to intersect and cannot be separated during the analysis of volumetric data due to lack of sufficient space for vertical growth, limiting the resolution in the *z* coordinate. Therefore the method we present is designed to process the data in 2D – as frequently done in the analysis of this type of data. To successfully separate distinct neuronal arbors, NeuroTreeTracer combines an automated method for soma detection and extraction that relies on multiscale directional filters, and a novel centerline tracing routine that identifies the neurites associated with each individual neuron using a front-propagation approach initiated from each soma location. *In addition to identifying the neuritic branches belonging to each neuron in a multicellular image*, *for each neuron NeuroTreeTracer labels its sub*-*compartments*, *i*.*e*., *soma*, *dendrites and axon*, *and determines the paths connecting soma to neurites*, *hence enabling the computation of geometrical characteristics and the quantification of local expression levels of analytes of interest with respect to their location along the neurites*.

As indicated above, NeuroTreeTracer is motivated by applications of neuron screening and profiling where fluorescence-based multispectral imaging is used to probe for the localization and distribution of molecules at the single-cell resolution level over cellular networks. To illustrate and validate the capabilities of our approach, we have applied NeuroTreeTracer to several confocal images of neuronal cultures, by successfully extracting and labelling individual neuronal trees in images containing up to 30 non-separated cells. As part of this work, we have also applied our algorithm to analyze the spatial distribution of ion channel complexes in a biologically meaningful context, namely, to quantify the redistribution of the native voltage gated Na^+^ channel complex at the AIS in response to alteration of the GSK3 pathway. NeuroTreeTracer is implemented in Matlab^19^ and is released open source and freely available to the scientific community.

## Methods

As indicated above, tracing algorithms available in the literature may be very effective in finding centerline traces in complex multicellular images. However they are not designed to identify and separate sub-traces associated with individual cells in a multicellular network. In this paper, we address the problem of extracting the labelled traces of each neuron in a multicellular image including their connectivity properties (what path connects a neurite location to its soma?). To emphasize the conceptual difference and clarify our terminology, we recall that in the mathematical language a *graph* is a network model consisting of a set of nodes joined by edges. By contrast, a *tree* is a special type of graph, where there is only one path between two nodes and a hierarchical structure^20^. We model a neuron as a *directed rooted tree*, that is, a hierarchical network model consisting of a special node called root (corresponding to the soma) and, for any other node, a single directed path to the root (corresponding to a neurite emanating from the soma). Hence our goal is to extract directed rooted trees in an image rather than to compute a generic graph. Each tree provides a local reference system for each individual neuron in a fluorescent image and will be useful to compute the spatial profile of molecular constituents of the neuron along its neurites.

As observed in the Introduction, the analysis of confocal image stacks in native 3D resolution is very challenging in images of cultured neurons due to the relatively small number of optical slices and to the poor image contrast of those optical slices farther away from the illumination source. As a consequence, image stacks are usually converted into 2D images by projecting the stack (comprising typically about 15–30 optical sections) along the axis perpendicular to the image plane (the *z* axis). The most common projections are the *average intensity projection* (AIP) that outputs a 2D image where intensity in each pixel is the average intensity in all voxels with the same (*x*, *y*) coordinates. Likewise the *maximum intensity projection* (MIP) is the 2D image wherein each pixel value is the maximum intensity of all voxels with the same (*x*, *y*) coordinate.

Our algorithm, NeuroTreeTracer, is designed to process 2D MIP images and consists of the following steps.

1. *Preprocessing*. Remove noise and improve image quality.
2. *Segmentation*. Separate neurons from background.
3. *Soma detection and extraction*. Find somas, identify soma regions and split somas that are clustered together.
4. *Tree extraction*. Extract the directed rooted tree associated with each individual neuron in the network.
5. *Computation of associative measures*. Compute local fluorescent intensity of individual neurons at the soma and along each neurite with respect to the arclength distance from the soma.

The key step and main novelty of NeuroTreeTracer is the computation of the tree structure of each neuron in an image. We describe below how we develop and implement each processing step, with emphasis placed in the last two steps containing the main original contributions of this paper.

### Proposed method

#### Data preprocessing

Images acquired through confocal microscopy are affected by several sources of degradation and need to be restored in order to facilitate the next processing steps, namely, segmentation and tracing. Such degradation includes the blurring due to the convolution of the original signal intensities with the point spread function of the imaging system and the noise introduced by the stochastic nature of the photon-counting process at the detector, which can be modeled as a Poisson-distributed random process. To restore the data, we adapt a denoising algorithm based on shearlets and adaptive thresholding, developed by some of the authors^21–23^. Unlike more traditional denoising methods, this approach is especially effective at preserving cell boundaries, since shearlet filters have highly anisotropic responses that are specifically designed to represent efficiently images with edges^21^ (www.math.uh.edu/~dlabate/software.html).

#### Segmentation

For this task, we adapt an algorithm recently developed by some of the authors that is based on Support Vector Machines (SVMs) and whose novel characteristic is the generation of features by a mix of multiscale isotropic Laplacian^24^ and shearlet directional filters^21^. As for many algorithms of this type, the proper classification stage of the algorithm is preceded by classifier training. This is the most computationally-intensive part of the algorithm, but it needs to be run only once as long as the segmentation algorithm is applied to images of the same type (e.g., same cell type and microscope setting). This routine, including the training stage, is fully automated and its performance is very competitive, as it was already demonstrated on multiple challenging 2D and 3D datasets in^5,25,26^. We refer the reader to the aforementioned references for more details about this approach.

#### Soma detection

The automated detection of soma location in fluorescent images is a challenging problem due to the lack of soma selective markers. In neuronal cultures, somas are usually visible in the channel marked by the MAP2 (microtubules associated protein 2) antibody staining, which is diffusely distributed in the entire somatodendritic compartment. As a consequence, further processing is needed to separate somas from dendrites. Conventional image analysis methods for soma detection frequently rely on binary masks generated from contrast enhancement and image intensity thresholding^27,28^. However this approach is not very effective when applied to fluorescent images since high intensity values are commonly found also outside somas. Therefore, in this paper, we apply a more sophisticated approach based on Directional Ratio, a multiscale geometric descriptor recently introduced by some of the authors in a prior work to overcome the limitations of conventional algorithms^26,29,30^. This method employs a collection of directional filters to compute, for each point in the image, a numerical score measuring the level of local anisotropy at a given scale. As shown in^26,30^, the application of this method is extremely effective to detect soma locations and, used in combination with the level set method or the fast marching approach, allows one to accurately and efficiently separate somas from neurites.

#### Tree extraction

The aim of this processing step is to compute a labelled rooted tree corresponding to each neuron in an image of a neuronal network. This requires to handle neurites that appear to cross or overlap and to determine the path connecting each neurite location to its respective soma.

Automatic tracing of neurons in fluorescent images is a challenging problem due to the topological complexity of the data and the irregularities of fluorescent signal intensity that may cause thin neurites to appear broken and neighbouring ones to merge. Several methods were proposed in the literature to address this task and the performance of existing methods may vary significantly depending on the quality of the image and the complexity of the structures to be recovered. For this reason, neuronal tracing is still an area of active investigation^31^. Among the existing methods, a number of algorithms compute traces through a process of skeletonization that may be applied to a smoothed version of the original image^32^; other methods have used various structural components to build-up the reconstruction by incrementally adding more and more such components into the morphological modeling of a neuron^6,7,33,34^; yet another class of methods rely on more sophisticated ideas to segment the image, next compute seed points and finally join the seeds to generate traces^5,35^. We refer to the excellent survey papers on neuronal tracing available in the literature^1,14,31^ for a more detailed critical discussion of existing methods.

A survey of the literature shows that existing tracing methods are typically designed to trace the entire network in the image so that – unless the image contains a single neuron or well-separated ones– they do not resolve the tree structures corresponding to individual neurons in the image in the sense discussed at the beginning of this section. Sorting out the neurites of each neuron from the graph of a neuronal network containing multiple (possibly non-separated) neurons would require splitting the graph by identifying all trees corresponding to individual neurons. This is a complex and challenging task that is not easy to perform automatically, in general, and we are not aware of any existing algorithm performing such task successfully. HCA-Vision^18^, for instance, computes a trace of a multicellular network including single-neuron traces *if neurons are separated in the image*. However, if neurons are not separated, neurites are assigned to a specific cell based essentially on proximity and without addressing connectivity properties. As a result, this method is unsuccessful in resolving the tree structure of individual neurons in general images. A further examination of this method in comparison to our approach is found in the Discussion.

Due to the difficulty of identifying and extracting the trees corresponding to individual neurons in a neuronal network by post-processing a fully traced network, we propose here a new tracing and sorting strategy. Our method will not attempt to trace every neurite in an image but only those neurites that are part of a neuronal tree, with the goal to recover every neuronal tree in the image. Starting from the soma locations we detected in the previous processing step, we will search for the trees associated to each neuron in the image by computing front-propagated traces originating from each soma. To carry out this task effectively, one major challenge is to resolve crossing and/or partially overlapping neurites. We describe below our step-by-step procedure for the extraction of neuronal trees from an image of a multicellular neuronal network. We assume that the binary segmented image and the soma masks are given as input. The routine consists of three steps: (i) initialization; (ii) seeding; (iii) tracing.

##### Step (i):

**Figure 1:**
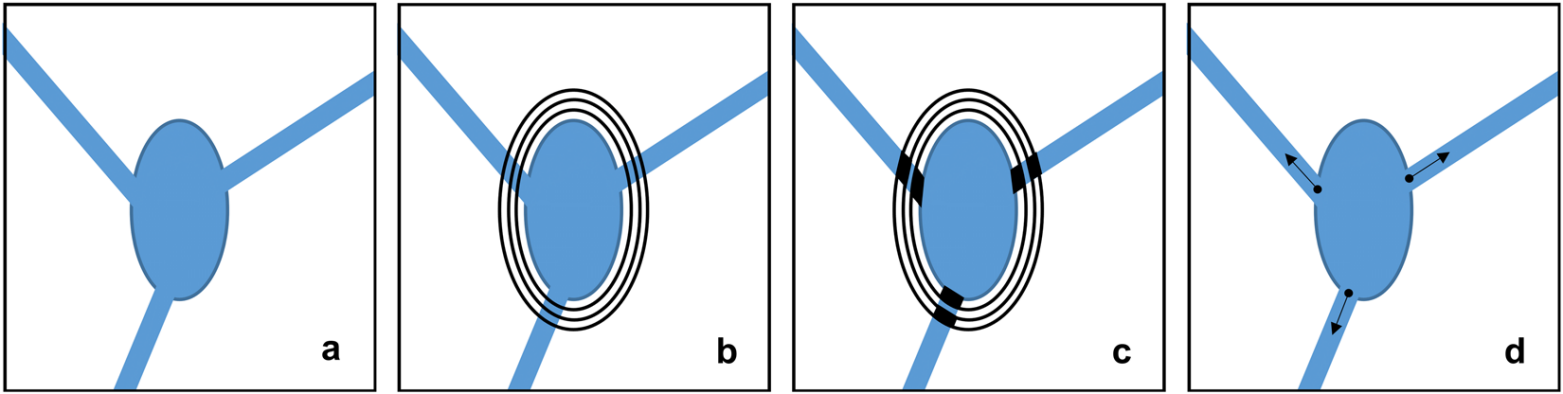
Tree initialization. (**a**) Idealized model of a binary segmented soma with 3 emanating neurites. (**b**) Soma boundary is dilated three times with increasing dilation factor and (**c**) the symmetric difference of the successive masks is intersected with the image producing the black regions shown in the panel. (**d**) By computing the centroids of the 6 black regions from panel (c) and then connecting the centroids located on the same branch with the soma, we find the starting location of each neurite and its initial orientation, as indicated by the black arrow.

*Initialization*. We apply successive dilation operators with rates *r* = 1.1,1.2,1.3 on each soma mask (Fig. 1b). Let us denote by *S*_0_ the soma mask and by *S*_*i*_, *i* = 1,2,3, the three dilated masks, ordered by increasing size. Next, take the symmetric differences *S*_1_Δ*S*_0_ and *S*_3_Δ*S*_2_ and their intersections with the segmented structures. For each neurite, this operation will identify two short neuritic segments in the proximity of the soma (Fig. 1c). Next we find the centroids of these regions and connect those located on the same neurite to its nearest soma mask *S*_*i*_. Thus we find the starting location of each neurite and its initial orientation, which is given by the orientation of the line connecting the centroids located on the same neurite (Fig. 1d).

##### Step (ii):

*Seeding*. We determine seeding points along the centerlines of the neurites by using an adaptation of a method in^5,25^.

For $x\in {{\mathbb{R}}}^{2}$, we define $Df(x)={{\rm{\min }}}_{y}\{\parallel x-y\parallel :f(y)=0\}$, where *f* is a binary segmented image. The local maxima of *Df* inside the structure are the points that are furthest away from the boundary of the neuron, since *f*(*y*) = 0 if *y* belongs to the image background. To enhance the magnitude of those local maxima and improve the robustness of the successive processing steps with respect to numerical rounding errors, the function *Df* is next convolved with the filter $$(\begin{array}{ccc}-{\textstyle \tfrac{1}{8}} & -{\textstyle \tfrac{1}{8}} & -{\textstyle \tfrac{1}{8}}\\ -{\textstyle \tfrac{1}{8}} & 2 & -{\textstyle \tfrac{1}{8}}\\ -{\textstyle \tfrac{1}{8}} & -{\textstyle \tfrac{1}{8}} & -{\textstyle \tfrac{1}{8}}\end{array})$$. After this step, we use a thresholding filter to select candidate seeding points along the centerline. Clearly, the lower the threshold, the more the seed points we derive. However, if the threshold value is too small, one may find more than one seed along the centerline resulting in irregular or inaccurate traces. On the other hand, if the threshold value is too large, then seeds may be very sparse and the distance between consecutive seeds might be so large that the tracing routine connecting potential seed points may terminate earlier than expected. We remark that the selection of the ‘best’ threshold value is dependent on the thickness and tortuosity of the neurite, so it is very difficult to determine this value automatically. Therefore, after seeds are generated using a reasonable threshold value (we set the value 0.16 in our experiments), we proceed as follows. For each generated seed *s*, we compute a ball centered at *s* with radius *Df*(*s*) and eliminate all other seeds found within this ball. If this process generates gaps along the centerline (when balls associated with different seed points do not intersect), then we generate additional seeds by computing again the distance function, *Df*, locally within that gap region, and then proceed as above. As demonstrated in^5,25^, this method is very reliable and competitive with respect to existing routines. We refer to those references for a more detailed discussion of this seeding strategy.

##### Step (iii):

*Tracing*. Starting from the initial location of a neurite found in Step (i), the algorithm searches for the closest seed location within a small search window whose goal is to favour the selection of points in the direction of the local orientation of the neurite. It then connects the two seed points. This process is repeated after each new seed is connected to the trace and it stops when no more seeds are located within each regarding window.

**Figure 2:**
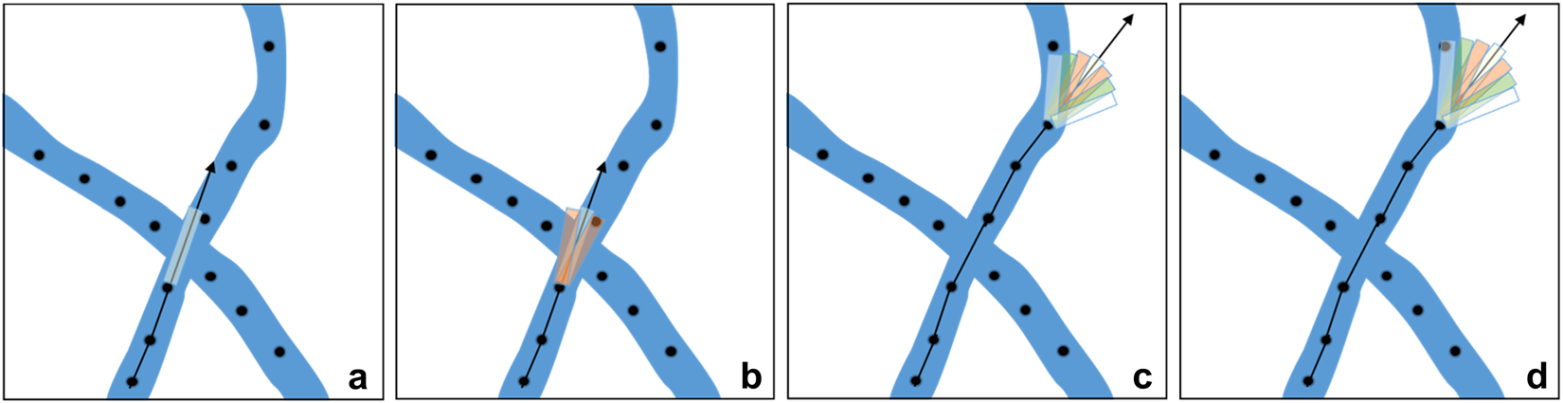
Tree tracing. (**a**) The search for the next node in the trace is initially restricted within a long rectangle whose main axis is oriented according to the local orientation of the neurite (black arrow). (**b**) If no seed is found, two additional rectangles are generated with orientations forming a small angle with respect to the local orientation of the neurite. This process ensures that the trace follows the given neurite and not the intersecting one. (**c**,**d**) If no seed is found within the approximately circular sector region, the search is repeated over a larger region obtained by increasing the length of the rectangular windows.

The search window plays a key role in this task, because it determines that the tracing continues on the same branch. When branches intersect in a maximum-intensity-projected image, the risk of switching to another branch becomes significant. The process for choosing where to continue is illustrated in panels (a and b) of Fig. 2. The main idea is that when neurites change orientation they do this in a smooth way. So abrupt changes of orientation of the tracing process are likely to lead a turn into a different branch. Hence, first the algorithm searches for the next seed within a long rectangular region whose long side is aligned with the expected orientation of neurite (Fig. 2a). For the initial location of each neurite, such orientation is estimated according to Step (i); for successive locations, the expected orientation of the neurite is estimated by measuring the direction of the two preceding seeds in the trace. If no seeds are found within this rectangle, then a pair of rectangular windows are generated with orientations forming a small angle with respect to the expected orientation of the neurite (Fig. 2b). The length of those new rectangles is slightly smaller than the previous rectangular region. This process continues (Fig. 2c) until either a seed is found or the orientation of the new rectangles exceeds a given angle (2*π*/5 in our experiments). This searching process is repeated multiple times generating every time a new approximate circular sector region of larger radius. That is, every time the searching process is repeated, longer rectangles are used to generate the new window region (Fig. 2d). If this process does not find a new seed after a number of attempts, we terminate the search and assume that the neurite is completely traced. This tracing routine is illustrated in Fig. 2.

In our numerical experiments we set the length of the initial rectangle to be 10 pixels. The searching process is repeated up to 10 times, every time increasing the length by 2 pixels.

#### Computation of fluorescent intensity profiles

The trace extracted in Step (iii) provides a spatial reference system to compute the local fluorescent intensity signal along each neurite. As the background intensity of a fluorescent image is typically non-zero, this background value needs to be subtracted in order to get a reliable measure of the fluorescent signal along a neurite. Furthermore, this value varies spatially and taking account of this spatial variability is critical to estimate fluorescent intensity values accurately. Therefore, to estimate the local value of background signal at a location near a neurite, we average the background signal computed on a pair of small windows (3 × 3 pixels) centered on a segment perpendicular to the neurite trace and displaced slightly away from the neurite (2 pixels away in our experiments). Since we have access to the segmented image, we can also ensure that such windows do not overlap existing structures (i.e., other neurites). Finally, the estimated background value is subtracted from the original fluorescent intensity value computed at the neurite location and the difference is the ‘true’ fluorescent intensity value estimated at the particular location.

### Cell preparation and imaging

The image datasets used in the present work are primary hippocampal neuronal cultures that were prepared in Dr. Laezza’s Laboratory at the Department of Pharmacology & Toxicology of the University of Texas Medical Branch. These images are part of a previously published set of data^36^.

Banker’s style hippocampal neuron cultures were prepared from embryonic day 18 (E18) rat embryos as described in previous work^36^. Following trituration through a Pasteur pipette, neurons were plated at low density (105 × 105 cells/dish) on poly-L-lysine-coated coverslips in 60 mm culture dishes in MEM supplemented with 10% horse serum. After 24 h, coverslips (containing neurons) were inverted and placed over a glial feeder layer in serum-free MEM with 0.1% ovalbumin and 1 mM pyruvate (N2.1 media; Invitrogen, Carlsbad, CA) separated by approx. 1 mm wax dot spacers. To prevent the overgrowth of the glia, cultures were treated with cytosine arabinoside at day 3 *in vitro* (DIV).

Hippocampal neurons (DIV14) were fixed in fresh 4% paraformaldehyde and 4% sucrose in phosphate-buffered saline (PBS) for 15 min. Following permeabilization with 0.25% Triton X-100 and blocking with 10% BSA for 30 min at 37 °C, neurons were incubated overnight at room temperature with the following primary antibodies: mouse anti-FGF14 (monoclonal 1:100; Sigma Aldrich, St Louis, MO), rabbit anti-PanNav (1:100; Sigma, St Louis, MO) and chicken anti-MAP2 (polyclonal 1:25000; Covance, Princeton, NJ) diluted in PBS containing 3% BSA. Neurons were then washed three times in PBS and incubated for 45 min at 37 °C with appropriate secondary antibodies as described for brain tissue staining. Coverslips were then washed six times with PBS and mounted on glass slides with Prolong Gold anti-fade reagent.

Confocal images were acquired with a Zeiss LSM-510 Meta confocal microscope with a 63X oil immersion objective (1.4 NA). Multi-track acquisition was done with excitation lines at 488 nm for Alexa 488, 543 nm for Alexa 568 and 633 nm for Alexa 647. Respective emission filters were band-pass 505–530 nm, band-pass 560–615 nm and low-pass 650 nm. Z-stacks were collected at *z*-steps of 1 *μ*m with a frame size of 512 × 512, pixel time of 2.51 *μ*s, pixel size 0.28 × 0.28 *μ*m and a 4-frame Kallman averaging. Acquisition parameters, including photomultiplier gain and offset, were kept constant throughout each set of experiments.

### Data availability

NeuroTreeTracer was implemented using MATLAB 7.12.0 (R2011a). The source code of the routines for neuron segmentation and soma detection was previously developed by some of the authors^30^ and is publicly available at the Github link: https://github.com/cihanbilge/SomaExtraction. The Matlab source code of the remaining routines are publicly available at https://github.com/cihanbilge/AutomatedTreeStructureExtraction. The imaging data used to validate the code are included with the software package.

## Results

In this section, we illustrate the application of NeuroTreeTracer for the extraction of labelled tree structures and the computation of local fluorescent intensity measures on a multiplicity of confocal images of neuronal cultures. Imaging data were generated by Dr. Laezza from the Department of Pharmacology & Toxicology at the University of Texas Medical Branch.

### Validation: neuronal tracing

**Figure 3:**
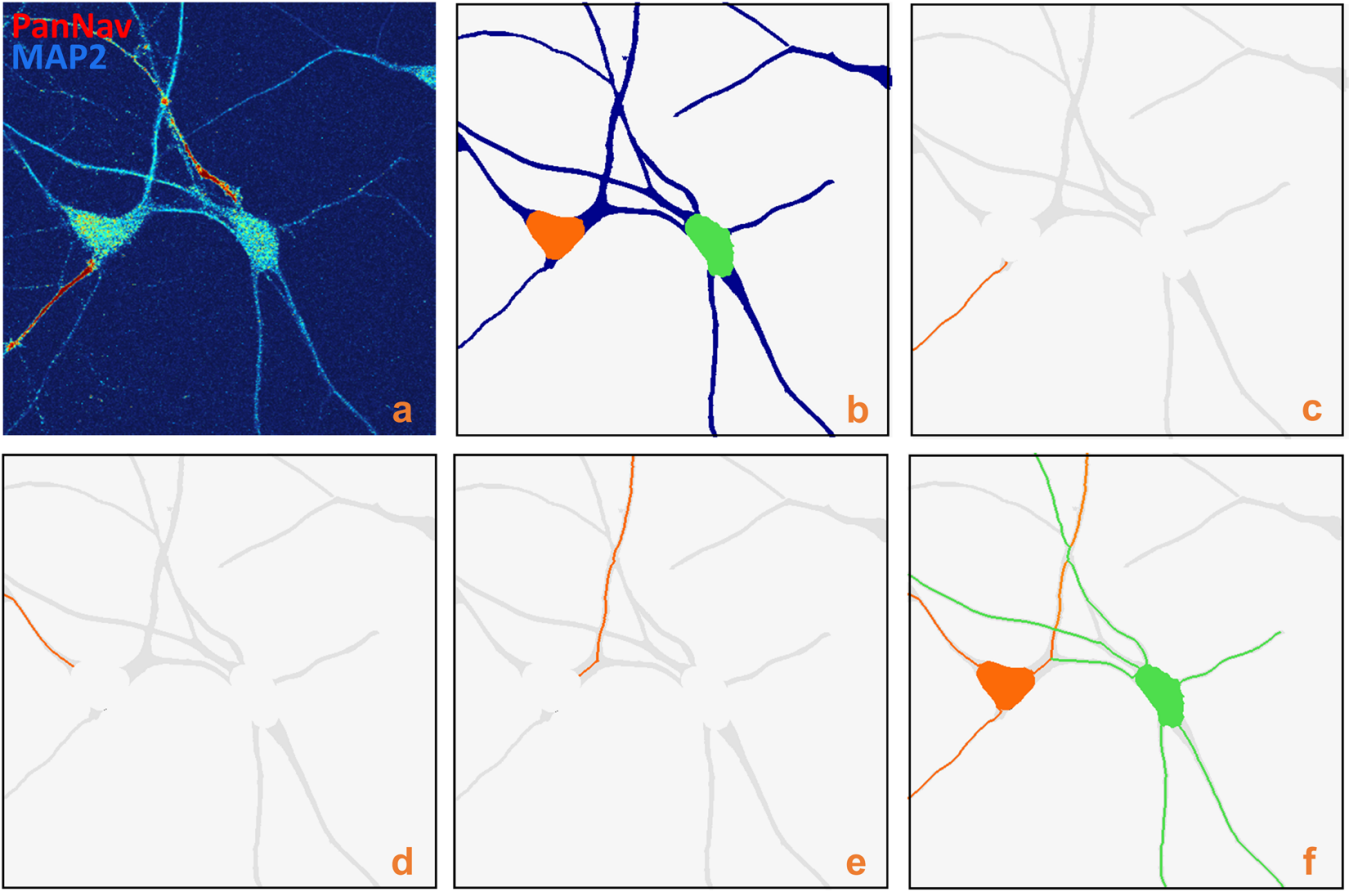
Neuron tracing. (**a**) Confocal image of cultured neurons (MIP view) labelled with an anti-Nav *α* subunit-specific antibody, PanNav, visualized with an Alexa 568 conjugated secondary antibody (red) and an anti-MAP2 antibody, visualized with an Alexa 647-conjugated secondary antibody (blue). (**b**) Corresponding segmented image with detected somas. (**c**–**e**) Starting from each soma location, the algorithm computes the centerline traces of each neurite. It correctly traces neurites even in the presence of crossing branches (panel (e)). (**f**) The tree structure of the network is completely solved by computing separate trees for each neuron, shown in different colors.

**Figure 4:**
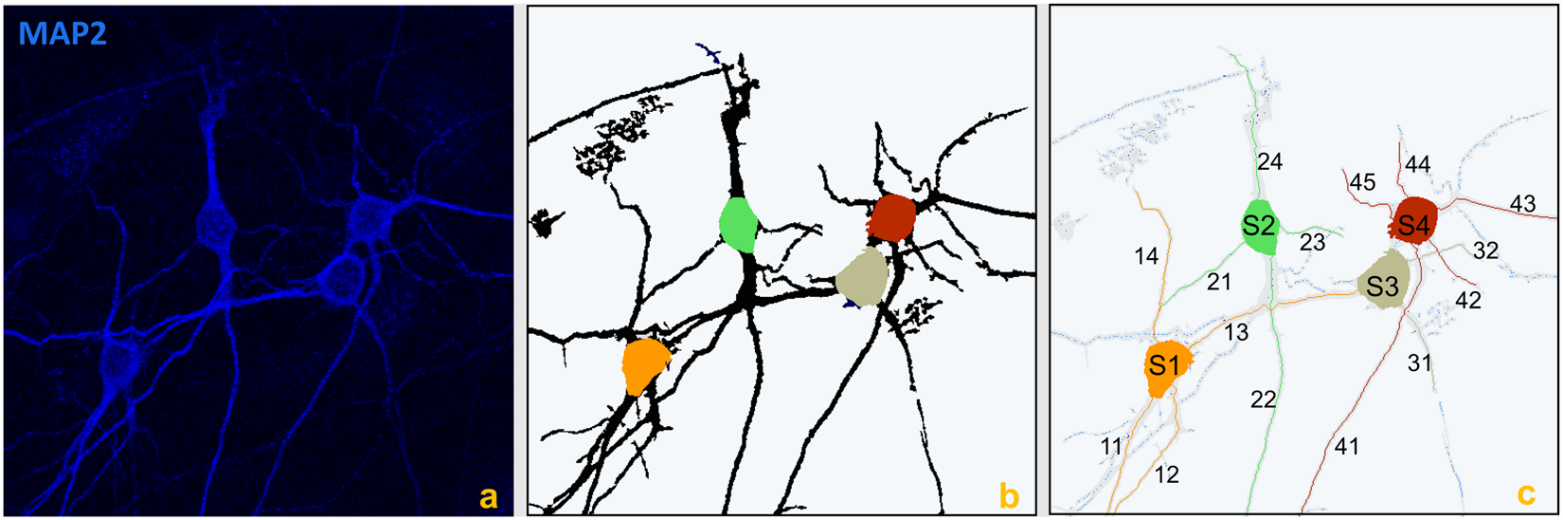
Neuron tracing. (**a**) Confocal image of cultured neurons (MIP view) labelled with MAP2 (blue) marker. (**b**) Corresponding segmented image with detected somas and (**c**) individual neuronal trees, automatically extracted and labelled. The algorithm successfully resolves most crossing points.

The images we considered for the first set of numerical experiments are confocal images of neuronal cultures containing between 2 and 8 neurons. Representative illustrations of the proposed neuronal tracing algorithm are shown in Figs 3 and 4. In particular, Fig. 3 displays several steps of the tracing routine showing that our algorithm correctly resolves crossing neurites and is able to assign each neurite to its corresponding cell. Figure 4 has a higher number of cells and a more complex topology. Also in this case, NeuroTreeTracer is able to resolve intersecting neurites. However, some neurites are not completely traced and the labeling of some neurites is ambiguous even for a manual annotator. In fact, in the situation of crossing neurites from several cells, our criterion for the assignment of each neurite to a specific neuron may be inconclusive as different branches may be associated with a similar change of orientation at the intersection point. Another potential source of ambiguity comes from incorrect or missed seed points. The performance of our tree extraction routine is clearly dependent on the performance on the seeding routine. This routine may fail to generate seeds in the correct locations near intersecting branches as they appear merged together and they may generate a blob-like region in the segmented image. As a result, the tracing routine may stop before a neurite is completely traced. We found that this situation is rare in the images we considered but it is a potential cause of errors in images containing a denser population of neurons.

Despite these shortcomings, the overall performance of NeuroTreeTracer is very satisfactory in the typical images of neuronal cultures we considered for this study. To assess the performance of the algorithm with respect to the ability to correctly trace a neurite and attribute it to the correct cell, we tested 12 images, each containing between 2 and 8 neurons, for a total of 49 neurons. As a performance metric we used the *accuracy* that is defined as *the ratio of the correctly traced and labelled neurites over the total number of neurites in a cell*. The correct traced and labelled neurites were determined based on the visual evaluation of domain experts.

#### Performance

Results summarized in Table 1 show the performance of NeuroTreeTracer on our entire set of test image using the standard performance metrics of precision, recall and dice coefficients^37^. The *Sensitivity* (or True Positive Rate or recall) measures the proportion of correctly identified neurites with respect to the total number of neurites (that are manually identified by a domain-expert without knowledge of the algorithm results). Denoting by *T* *P* (= true positive) the number of correctly detected and identified neurites and by *F* *N* (=false negative) the number of missed neurites, we define:

1 $${\rm{Sensitivity}}=\frac{TP}{TP+FN}.$$

**Table 1:** Performance analysis using our algorithm NeuroTreeTracer and HCA-Vision on a set of 12 confocal images containing a total of 49 neurons and 191 neurites.

	Number of neurites	Sensitivity	Precision	Dice coefficient	Crossing
NeuroTreeTracer	191	0.90	1.00	0.94	74%
HCA-Vision	191	0.85	0.49	0.62	20%

The *Precision* measures the proportion of correctly identified neurites over all detected neurites. That is, denoting by *F* *P* (= false positive) the number of neurites detected but wrongly identified,

2 $${\rm{Precision}}=\frac{TP}{TP+FP}.$$

Finally, the *Dice coefficient* is useful to compare the similarity between two measures and is given by

3 $${\rm{Dice}}\,{\rm{coefficient}}=\frac{2\,TP}{2\,TP+FN+FP}.$$

The dice coefficient can be considered as a measure of the overall effectiveness of the neurite extraction algorithm.

The table shows that NeuroTreeTracer performs very well with respect to all metrics (the closest to 1 the better).

### Validation: neuronal tracing on larger images

**Figure 5:**
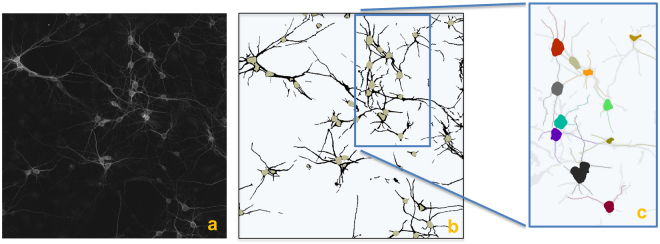
Tree extraction on a large tiled and stitched image. (**a**) Tiled and stitched confocal fluorescent image (MIP view) of a neuronal culture. Image size = 1894 × 1894 pixels (1 pixel = 0.28 × 0.28 *μ*m). (**b**) Segmentation and soma detection. (**c**) Extraction of neuronal trees on a representative subregion (blue box). The algorithm is ignoring those cells whose soma is overlapping the box boundary.

A natural question is about the applicability of NeuroTreeTracer to images containing a larger number of neurons. To show how our method performs in this situation, we applied NeuroTreeTracer to a tiled and stitched fluorescent image of a neuronal culture containing about 40 neurons. As the processing time depends on the number of neurons contained in the image (the current algorithm generates each neuronal tree sequentially), to speed up the computation we can partition the segmented image into partially overlapping rectangles and process each sub-image separately and in parallel. As each rectangular window can be processes separately and the results successively combined, this provides a viable and computationally efficient strategy to process large images. To determine such rectangular sub-images, we proceed by listing the somas (already segmented and labelled), collecting them in subsets based on proximity and then partitioning the image into rectangles, each one containing only a subset of the somas. In doing this, we ensure that each soma in a given subset is fully contained in the rectangle. If a soma from another subset overlaps the boundary of the rectangle, it would be ignored. The application of this idea is illustrated in Fig. 5, showing that the large image is segmented and the somas are detected; next the extraction of neuronal trees is applied within a rectangular window inside the image.

### Application: neuron profiling

One main motivation for the development of NeuroTreeTracer comes from applications in image-based neuron profiling, where it is critical to quantify morphological changes of neurons and alterations in the expression levels of their molecular constituents at the single-cell level.

To illustrate the potential of our approach in such studies, we have applied NeuroTreeTracer to the analysis of a set of confocal images of neuronal cultures where primary mouse hippocampal neurons had been exposed to an inhibitor of the glycogen synthase kinase 3 (GSK3) pathway. As observed by one of the authors, inhibition of GSK3 correlates with alterations in the distributions of critical molecular constituents of the axonal initial segment (AIS), including subcellular redistribution of the native voltage gated Na^+^ (Nav) channel complex^36^. NeuroTreeTracer offers an ideal platform to precisely quantify such alterations in an image, as it generates a spatial reference system of each individual neuron that can be used to measure the intensity values of fluorescent signal along each neurite with respect to the arclength distance from the soma. These measures generate classifying features associated to individual neurons exposed to specific perturbations.

**Figure 6:**
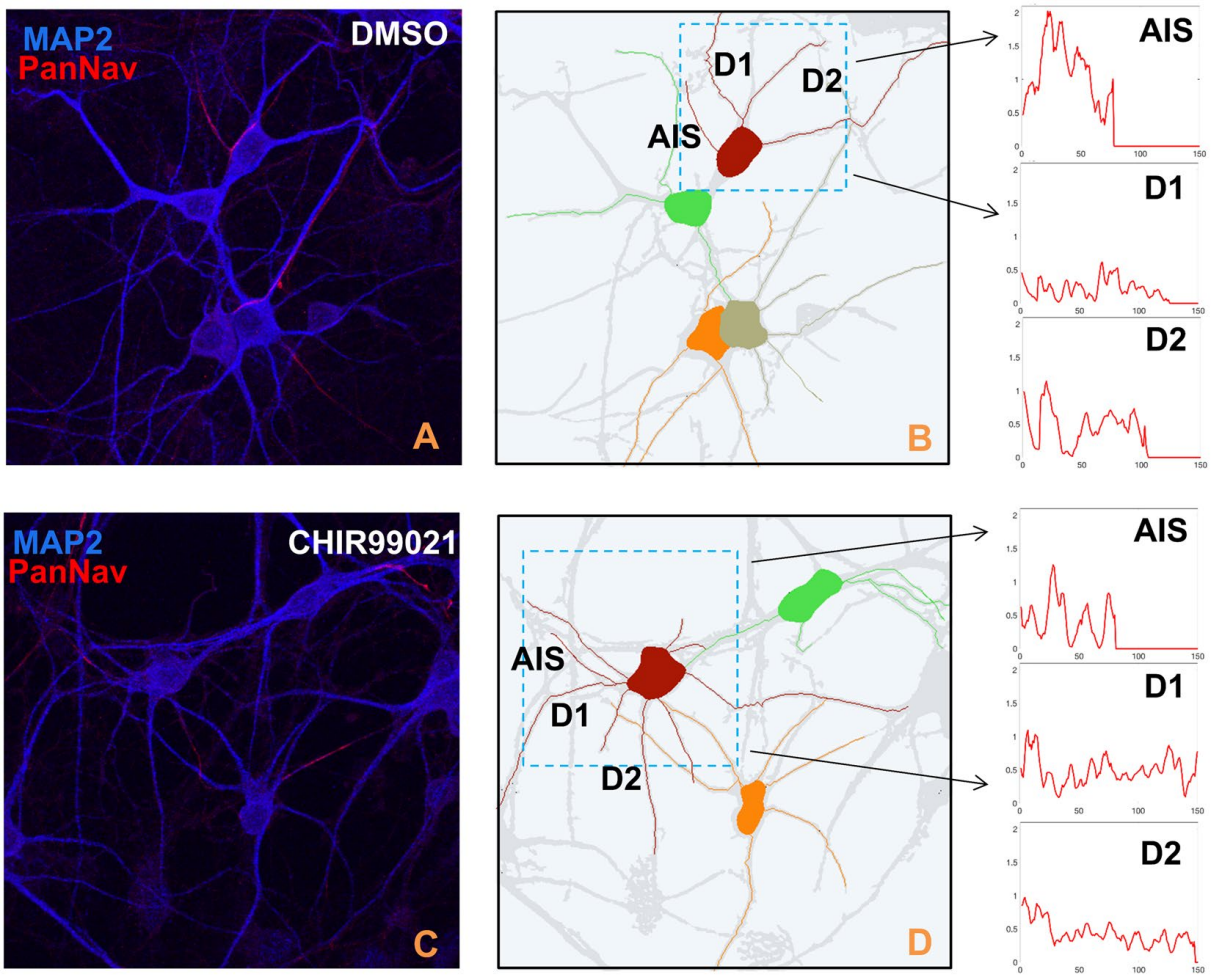
Inhibition of GSK3 leads to redistribution of AIS proteins. Representative confocal and corresponding traced images of hippocampal neurons in control (DMSO) condition (**A**,**B**) or following inhibition of GSK3 induced by CHIR99021 (**C**,**D**). Neurons are labelled with PanNav (red) and MAP2 (blue) markers. Fluorescence intensity values corresponding to the red channel along the AIS and dendrites (D1 and D2) in control (top right) and CHIR99021 (bottom right) are computed using traces in panels (b,d). Note the loss of the bell-shaped cluster distribution of Nav channels treated with the GSK3 inhibitor compared with the control group.

Using this method, we have analyzed 10 confocal images of neuronal cultures associated to two experimental groups – one group involving neurons exposed to CHIR99021, an inhibitor of GSK3, and the other group for the control case (DMSO treated). As indicated in the Methods section, these images are part of a previously published set of data^36^. In total, we have extracted individual neuronal trees of over 30 neurons and computed the fluorescent intensity profiles along their neurites. Figure 6 illustrates the outputs of our algorithm on two representative confocal images: one image contains control neurons exposed to DMSO (panels (a–b)) and another image contains neurons exposed to CHIR99021 (panels (c–d)). The plots of fluorescent signal in panels (a) and (d) indicate a reduced expression level of the Nav channel complex at the AIS in the CHIR99021 neurons with respect to DMSO.

**Figure 7:**
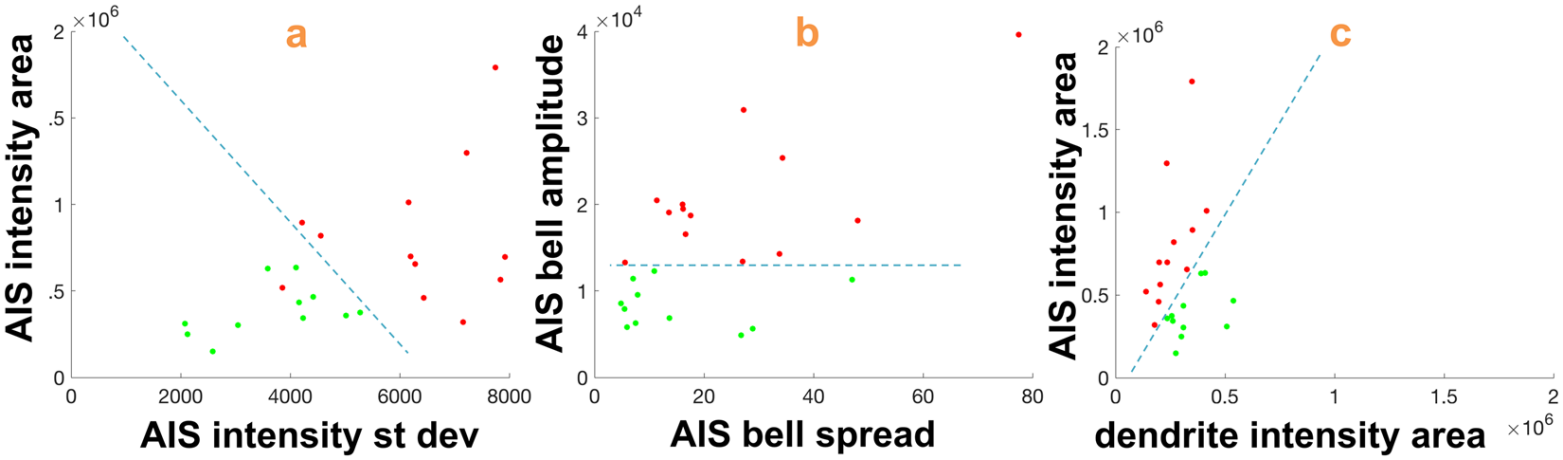
Pattern of subcellular distribution of the Nav channel complex. For all scatterplots, green dot = CHIR99021-inhibited neuron, red dot = DMSO neuron. (**a**) Relationship between the area of the fluorescent intensity signal measured at the AIS and the standard deviation of the signal measured over the same interval 0–70 pixels. (**b**) Relationship between the AIS bell-shaped amplitude at the AIS using a Gaussian approximation (as described in text) and standard deviation of approximating function. (**c**) Relationship between the area of the fluorescent intensity signal measured at the AIS and the area of the fluorescent intensity signal measured along the dendrites over the same interval 0–70 pixels (1 pixel = 0.28 *μ*m). For all plots, the dashed blue line is drawn to show separation or approximate separation between the two classes.

Complete results of our analysis are reported in Fig. 7. Of over 30 neurons initially traced, 23 neurons were selected for further analysis (12 DMSO, 11 CHIR99021) as we excluded those neurons for which no axon was visible (since located outside the image). To quantify the *heterogeneity* between perturbed and control neurons at the AIS, we considered several quantities: the area *A*_*AIS*_ of fluorescent signal intensity at the AIS (70 pixels in length, 1 pixel = 0.28 *μ*m), the variance *V*_*AIS*_ of the signal over the same interval and the *AIS bell*-*shaped amplitude H* obtained by approximating the fluorescent intensity profile at the AIS with a Gaussian function $g(x)=H\,\exp (-\frac{{(x-\mu )}^{2}}{2{\sigma }^{2}})$ (approximation is meant in the standard least squares sense). We found that with respect to all such quantities the difference between CHIR99021 and DMSO neurons is statistically significant, with the DMSO neurons showing larger values of *A*_*AIS*_, *V*_*AIS*_ and *H*. The significance was measured using a two-sample *t*-test with significance level *α* = 0.05 and the computed two-tail p-values are 0.00444, 0.00015 and 0.00005 for *A*_*AIS*_, *V*_*AIS*_ and *H* respectively. Note that our newly introduced quantity *H* has the smallest p-value and it (linearly) separates the two classes of neurons (Fig. 7b); this is not true for the other measures (Fig. 7a). To quantify *polarity* in the data, we computed the ratio *R*_*AD*_ of the area *A*_*AIS*_ of fluorescent signal intensity at the AIS vs. the dendrite area *A*_*den*_. The dendrite area *A*_*den*_ is obtained by averaging the fluorescent signal intensity along two dendrites whose thickness is comparable to the AIS, over the same length of 70 pixels. We found that the difference of the value *R*_*AD*_ between CHIR99021 and DMSO neurons is statistically significant (two-tail p-value = 0.00007), with the CHIR99021 neurons showing a value close to 1 and the DMSO neurons showing a value close to 3 (Fig. 7c). All results are consistent with the manual analysis carried out in previously published work^36^.

### Computation time, hardware and software

We implemented our routines using MATLAB 7.12.0 (R2011a). The numerical tests were performed using a MacBook with Intel Core i5 2.4 GHz processor and 16 GB RAM. On a 2D image of size 512 × 512 pixels, the average computing time for the shearlet-based denoising was approximately 7 seconds; the average computing time for the 2D segmentation routine was approximately 8 seconds; the average computing time of the soma segmentation routine was approximately 5 seconds; the average computing time of the tracing routine for a cell containing 3 branches was approximately 130 seconds. Note that our soma segmentation routine includes subroutines implemented in C++ to improve computational efficiency, as discussed in^30^. The tracing routine was not optimized for computational efficiency and its computing time could be reduced by implementing some subroutines in C++ and precomputing some filters.

## Discussion

This paper introduces a novel image processing pipeline called NeuroTreeTracer that is designed to extract individual neuronal trees from images of multicellular networks where neurons are not necessarily separated. Existing neuronal tracing algorithm in the literature are typically designed to process images containing a single neuron or to trace an entire image without sorting traces into arborizations corresponding to individual neurons. Breaking up the trace of a multicellular network into single-neuron tree is a very challenging task in general as it would require to solve a complex sorting problem. NeuroTreeTracer addresses this task by re-designing the tracing process. After detecting each soma in an image containing multiple neurons, the algorithm discovers each neuronal tree by computing a directed path for each neurite starting from its soma and resolving the connectivity properties of neurites that appear to cross or overlap.

**Figure 8:**
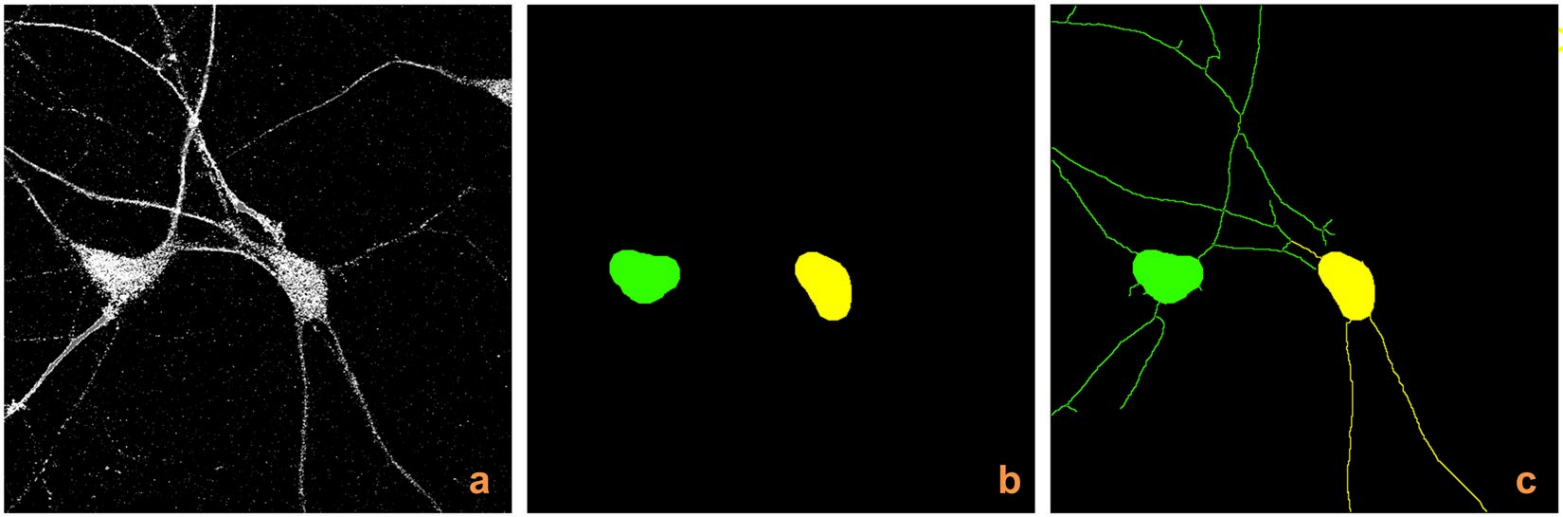
Neuron tracing using HCA-Vision. (**a**) Confocal image of cultured neurons (MIP view) labelled with MAP2 (blue) marker. (**b**) Detected somas and (**c**) traced image where cells and corresponding neurites are shown in same color. Note that the algorithm is unable to resolve intersecting neurites.

For comparison of our approach with existing tracing algorithms that also handle image with multiple neurons, we tested the algorithm HCA-Vision^18^. It is a popular software for high-content analysis that is available as freeware and that is very similar to routines included in the Cellular Imaging and Analysis commercial software of PerkinElmer. It is designed to automatically trace images of cultured neurons and compute morphometric parameters of their neurites but it is not designed to extract individual neuronal traces unless neurons are separated. As the authors write, “only isolated neurons having no contact with other neurons” can be analyzed and if neurons are not isolated “the decision to attribute a neurite touching two cells to either of them is arbitrary.” In addition, the method does not output a path connecting each neurite to the corresponding soma. As shown in Fig. 8, HCA-Vision is unsuccessful in tracing intersecting neurites in a rather simple image where instead our method was very successful (cf. Fig. 2). For a more general assessment, we compared the performance of NeuroTreeTracer and HCA-Vision using our entire set of test images with respect to the ability to attribute a neurite to the corresponding neuron and to solve intersecting neurites. Results reported in Table 1 show that HCA-Vision has significantly lower precision and dice coefficients than NeuroTreeTracer on the images we considered due to its limitations in assigning neurites to the correct neuron. We also include in the table the percentage of crossing neurites that are solved correctly, which is only 20% for HCA-Vision (as we wrote, the method is not designed to handle such locations) as compared to 74% for our method.

In summary, the results reported in this paper show that NeuroTreeTracer is highly reliable in resolving individual neuronal trees in confocal images containing multiple neurons even when they are not separated. Our algorithm is aimed primarily at large field-of view multispectral confocal images of neuronal cultures and is motivated by applications in neuron screening and profiling where it is important to measure the location and spatial distribution of molecules at the single-cell resolution level. For such applications, it is required to extract individually labelled neuronal trees and the paths connecting each neurite to the corresponding soma.

To illustrate the potential of NeuroTreeTracer for these applications, we have examined to a set of confocal images of neuronal cultures comprising two experimental groups, namely neurons exposed to an inhibitor of the GSK3 pathway and control. Using our NeuroTreetraces, we found each neuronal tree in the images and used this local reference system to measure the local fluorescent intensity profiles along the neurites with respect to the arclength distance from the corresponding soma. These measures – used as features for each cell – reveal that GSK3 inhibited neurons are associated with subcellular redistribution of the native Nav channel complex, confirming previously published results. With respect to the manual analysis carried out in previous studies, NeuroTreeTracer automatically generates fast-to-compute and reliable fluorescent intensity measures where local background noise is automatically removed. The flexibility of our computational platform also provides the ability to define novel measures of subcellular distributions of analytes of interest such as the AIS bell-shaped amplitude we introduced above. This quantity is be a novel measure of heterogeneity of the AIS that appears to be more robust than other more conventional measures.

Even though NeuroTreeTracer was designed to process confocal images of neuronal cultures, the ideas proposed and applied here are applicable to other types of imaging data. In particular, our segmentation routine has been tested on other microscopy images and image stacks^5,25^. Similarly the design of our soma detection, seeding and tracing routines are expected to work equally well on other types of microscopy images. As discussed above, the main limitation of the proposed tracing routine is that, as the density of cells increases and they become more clustered, it might be increasingly more difficult to solve intersecting neurites and attribute them reliably to a single cell. A possible way to mitigate this problem would be to allow a domain-expert user to manually address conflicts by possibly taking advantage of additional information, e.g., physiology, prior-knowledge, etc.

Finally, the ideas presented in this work are expected to apply to three-dimensional data. The preprocessing, segmentation and soma detection steps of the algorithm have already been developed and applied to volumetric data^5,22,26^. Our method for tree extraction routine in 2D can be adapted to the 3D setting. Indeed, the tree extraction would be easier for volumetric data, as neurites from different cells are not expect to crossover.
